# Transcriptomic analysis of developing sorghum grains to detect genes related to cell wall biosynthesis and remodelling

**DOI:** 10.1186/s12863-024-01198-x

**Published:** 2024-02-07

**Authors:** Camille Costes, Sergi Navarro Sanz, Caroline Calatayud, Alexandre Soriano, Hamza Mameri, Nancy Terrier, Mathilde Francin-Allami

**Affiliations:** 1 INRAE, UR1268 BIA Nantes, France; 2 CIRAD, UMR AGAP Institut, 34398 Montpellier, France; 3 CIRAD, INRAE, UMR AGAP Institut, Univ Montpellier, Institut Agro, Montpellier, France; 4 Montpellier Ressources Imagerie, BioCampus, University of Montpellier, CNRS, INSERM, Montpellier, France; 5 UMR IATE, Univ Montpellier, INRAE, Institut-Agro Montpellier, 34060 Montpellier, France

**Keywords:** Sorghum, Grain development, Laser microdissection, Transcriptome, Cell wall polysaccharides

## Abstract

**Objective:**

Sorghum (*Sorghum bicolor* (L.) Moench) is the fifth most important grain produced in the world. Interest for cultivating sorghum is increasing all over the world in the context of climate change, due to its low input and water requirements. Like other cultivated cereals, sorghum has significant nutritional value thanks to its protein, carbohydrate and dietary fiber content, these latter mainly consisting of cell wall polysaccharides. This work describes for the first time a transcriptomic analysis dedicated to identify the genes involved in the biosynthesis and remodelling of cell walls both in the endosperm and outer layers of sorghum grain during its development. Further analysis of these transcriptomic data will improve our understanding of cell wall assembly, which is a key component of grain quality.

**Data description:**

This research delineates the steps of our analysis, starting with the cultivation conditions and the grain harvest at different stages of development, followed by the laser microdissection applied to separate the endosperm from the outer layers. It also describes the procedures implemented to generate RNA libraries and to obtain a normalized and filtered table of transcript counts, and finally determine the number of putative cell wall-related genes already listed in literature.

**Supplementary Information:**

The online version contains supplementary material available at 10.1186/s12863-024-01198-x.

## Objective

Sorghum (*Sorghum bicolor* [L.] Moench) is a "C4" cereal whose interest in agricultural practice lies mainly in its resistance to drought and high temperatures [1]. In addition, the attraction of sorghum grain for human consumption is growing, due to its gluten free and its richness in health promoting macro and micro-nutrients [2]. Cell walls are an important variable in nutritional quality of grains as a dietary fiber intake. They are mainly composed of polysaccharides, whose composition may differ greatly between endosperm and outer layers. Our objective is to gain a better understanding of cell wall polysaccharide deposition during grain filling. For this, we monitored whole gene expression both in endosperm and outer layers during grain development to identify those who could be involved in cell wall biosynthesis and remodelling. While several works focused on the composition of the cell walls of dry mature grains [3–6], little research has concentrated on the development of sorghum grains [7, 8]. The transcriptome of developing grains focusing on cell wall genes has been established for cereals such as barley [9], wheat [10] or rice [11]. In developing sorghum grains, transcriptomic analyses have focused mainly on starch, dhurrin and tannins [1, 12–14]. Transcriptomic analyses specifically targeting the cell wall gene set have been carried out mostly on sorghum stem [15, 16]. Our transcriptomic results will be combined with analyses of polysaccharide contents to reach a comprehensive view of cell wall assembly during sorghum grain development.

## Data description

In this study, MACIA, a sorghum cultivar with white grain without tannin, was used. The plants were sown and grown in greenhouses at CIRAD Montpellier in 2022 according to the conditions described in Table 1 [17]. The grains were harvested in triplicate at 7, 13 and 30 days after flowering (DAF), covering the beginning of development until near maturity, frozen in liquid nitrogen and stored at -80°C before microdissection step. All the following experiments were carried out under RNAse-free conditions. Grains were embedded as blocks in O.C.T (VWR® Q Path® O.C.T. Compound Mounting Medium, PA, USA) using liquid nitrogen to harden the medium [18]. From these blocks, sections of 18 µm were cut using a Cryostat microtome (MICROM HM 520, Leica, IL, USA) before to be adhered to frame slides (steel frame, PET-membrane 1.4 μm; RNAse-free.4 μm; RNAse-free, Leica, Germany). The sections were fixed on the slide by immersion in a 50 mL tube of iced pure ethanol for 30s. Then, they were immersed for 4 min in a 50 mL tube of iced 70% ethanol to remove O.C.T. Finally, the sections were dried at 40°C for 10 min [19]. Once the slides had dried, outer layers and endosperms were separated by laser microdissection using a Leica LMD7000 laser (Laser parameters: Power 50, Speed 6, Aperture 14, Pulse Frequency 1260, Specimen Balance 12) as previously described [20]. Samples were collected in 50 µL tubes (RNAse-free) containing cell lysis buffer (Nucleospin RNA, mini kit for RNA purification, Macherey–Nagel, ref 740,955.50, Germany). The tubes were then frozen in liquid nitrogen and stored at -80°C for storage prior to RNA extraction. Total RNA was extracted following instructions of the NucleoSpin RNA extraction kit (Macherey–Nagel, Germany). RNA quantity and quality were evaluated by automated electrophoresis (4200 Tapestation system, Agilent Technologies, CA, USA). RNA libraries were prepared according to the Illumina protocol with the TruSeq RNA Library Prep Kit (Illumina, CA, USA). The indexed libraries were pooled in 18-plex and subjected to pair-end 2 × 150 bp sequencing on an Illumina HiSeq2500 (GENEWIZ, By Azenta Life Sciences, Germany). The raw sequence files are available on the short-read archive under the bioproject PRJNA1029354 [21]. The software Cutadapt (version 3.5) [22] was used to eliminate low-quality sequences with a cut-off of 30, remaining adapters, as well as resulting reads shorter than 35 bases. Reads were then mapped across the entire sorghum Sbicolor_313_v3_2 genome using HISAT2 [23, 24]. Read count was then performed using featureCounts (from subread 2.0.1 [25]) with the parameters -M –fraction to count multimapping reads. In our study, reads were aligned to 34129 transcripts among 34496 genes of the sorghum genome (Sbicolor_453_v3.2).

**Table 1:** Greenhouse growing conditions for sorghum

Greenhouse conditions	Day	Night
Time	12 h	12 h
Temperature	28°C	22 °C
Sunlight	Natural light complemented by artificial led light (700 μmol m^–2^ s^–1^)	
Moisture	55% average, up to 75% maximum	

**Fig. 1:**
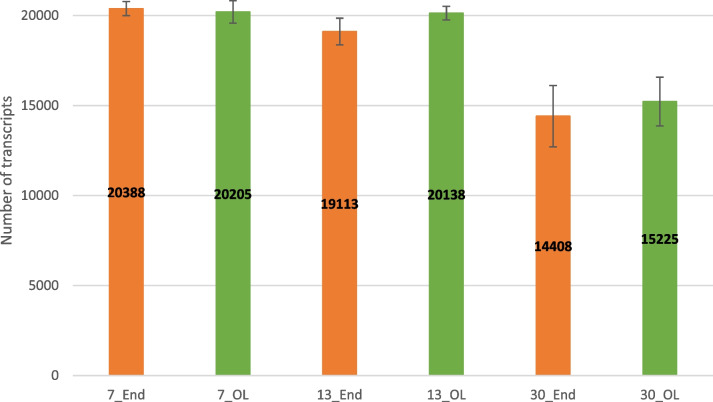
Number of transcripts expressed in each sample. 7_End: endosperm at 7 DAF (Days After Flowering); 13_End: endosperm at 13 DAF; 30_End: endosperm at 30 DAF; 7_OL: outer layers at 7 DAF, 13_OL: outer layers at 13 DAF, 30_OL: outer layers at 30 DAF. Data are the mean ± Standard deviation of three biological replicates

The DIANE interactive workflow was used to normalize the data with the TMM method (Trimmed Mean of M values) and to process them [26]. Only genes with a sum of reads under all conditions equal or greater to 100 were conserved, thus resulting to a final count of 20,710 transcripts. The average number of transcripts detected is higher at the early stages of development, at 7 DAF and 13 DAF, compared with the older development stage at 30 DAF (Fig. 1, [27]).

**Fig. 2:**
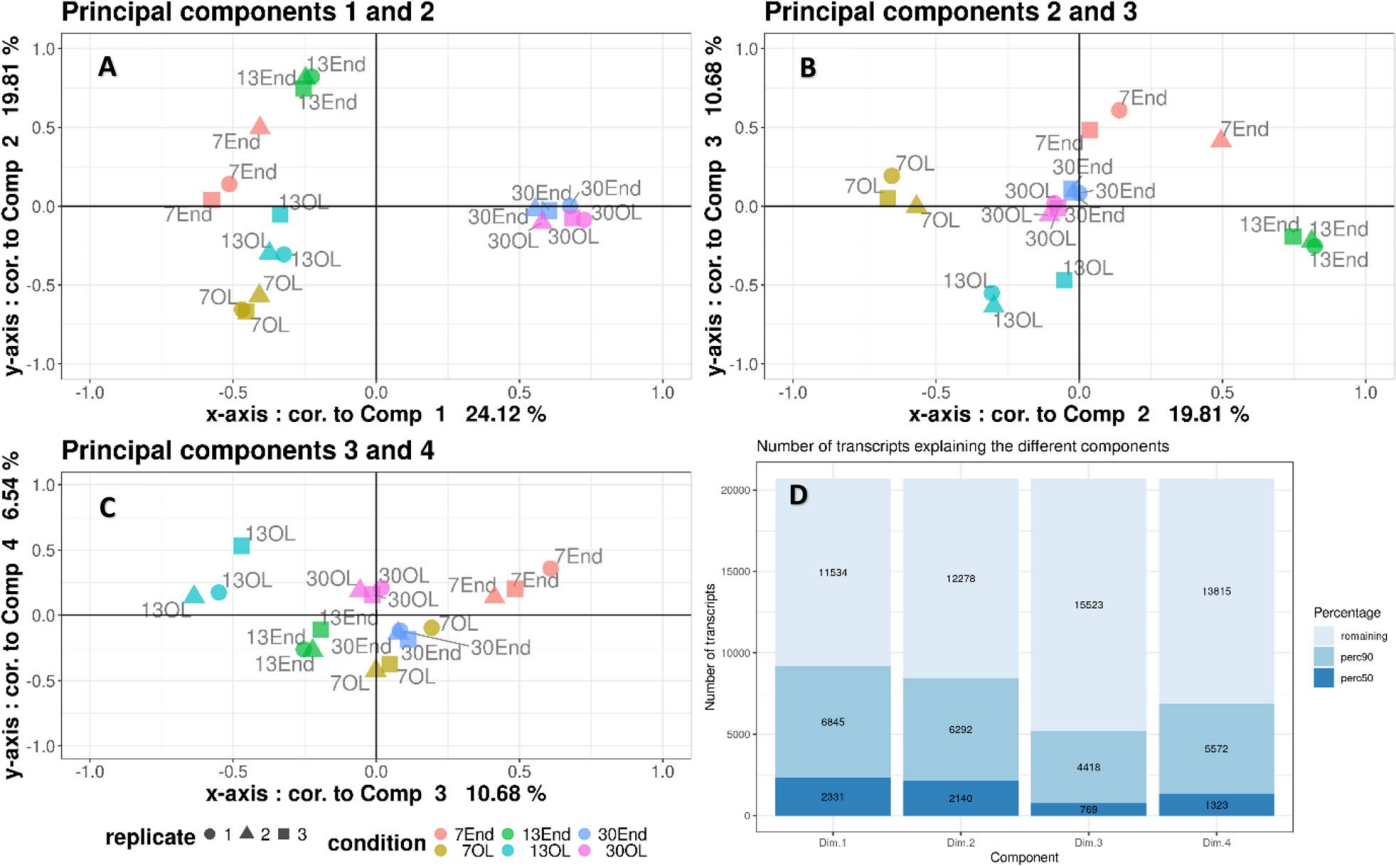
Principal component analysis (PCA) of transcriptomic data from endosperm (End) and outer layers (OL) of sorghum grains at different developmental stages (7, 13 and 30 Days After Flowering (DAF)). A, B, C: representation of samples as functions of components 1 to 4. D: Number of transcripts explaining 50% and 90% of the different components. 7_End: endosperm at 7 DAF; 13End: endosperm at 13 DAF; 30_End: endosperm at 30 DAF; 7_OL: outer layers at 7 DAF, 13_OL: outer layers at 13 DAF, 30_OL: outer layers at 30 DAF

The Principal Component Analysis (PCA) plot of the filtered and normalized RNA-seq data indicated that the biological replicates are well grouped. Components 1 and 2 account for 43.9% of the variance. The component 1 separates the early stages of development (7 and 13 DAF) from the near-mature stage (30 DAF). Tissues are separated from each other by component 2, although this is less obvious for the samples collected at 30DAF (Fig. 2, [28]). A list of 655 sorghum cell wall genes was previously constructed [15, 29]. Among them, 384 were detected in our filtered data from our different samples (Additional file 1, [30]). The quantifications of these cell wall genes, obtained for each biological stage, tissue and replicate, have been detailed in Additional file 1 [30]. These data will be thoroughly analysed and linked to future biochemical composition analyses.

### Limitations

During laser microdissection, the aleurone layer, made up of differentiated cells located between the endosperm and the outer layers, was mainly recovered with the outer layers. However, endosperm fractions could be contaminated by some aleurone cells during dissection.

### Supplementary Information

**Additional file 1.** List of the cell wall genes sorted from our transcriptomic analysis and their quantification.

## Data Availability

The raw sequence files are available on the short-read archive under the bioproject PRJNA1029354.

## Abbreviations

CIRAD: Centre de coopération internationale en recherche agronomique
DAF: Days After Flowering
RNA: RiboNucleic Acid
PCA: Principal Component Analysis
